# Clostridium Difficile and COVID-19: Novel Risk Factors for Acute Portal Vein Thrombosis

**DOI:** 10.1155/2021/8832638

**Published:** 2021-02-27

**Authors:** Venkata Ram Pradeep Rokkam, Gurusaravanan Kutti Sridharan, Rathnamitreyee Vegunta, Radhakrishna Vegunta, Umesha Boregowda, Babu P. Mohan

**Affiliations:** ^1^Internal Medicine, University of Arizona/Banner University Medical Center, Tucson, Arizona, USA; ^2^Internal Medicine, Westchester Medical Center, Valhalla, New York, USA; ^3^Department of Oncology, Sanford Health /University of North Dakota School of Medicine and Health Sciences, Fargo, USA; ^4^Internal Medicine, Bassett Medical Center, Cooperstown, NY, USA; ^5^Gastroenterology & Hepatology, University of Utah Health, Salt Lake City, Utah, USA

## Abstract

The COVID-19 pandemic has created an unprecedented global health care crisis. COVID-19 patients are found to have increased thrombotic risk. Despite being on prophylactic anticoagulation, many develop serious arterial and venous thromboembolic events. Emerging reports indicate COVID-19 may be considered a novel risk factor for portal vein thrombosis. Although, intra-abdominal infections are identified as risk factors, clostridium difficile colitis has not been typically seen as a risk factor for PVT. We report a case of an elderly female with a recent diagnosis of COVID-19 and no prior history of cirrhosis or malignancy who presented with diarrhea due to clostridium difficile infection. She developed sudden onset severe abdominal pain during the course of hospitalization. Acute portal vein thrombosis was identified on CT imaging of the abdomen, and she improved well with therapeutic anticoagulation. Acute portal vein thrombosis usually results from a combination of local and systemic prothrombotic risk factors. The combination of local infection by clostridium difficile and COVID-19 coagulopathy led to development of portal vein thrombosis in our patient. To the best of our knowledge, this is the first case of portal vein thrombosis reported in a patient with clostridium difficile infection in the setting of COVID-19 coagulopathy. During the current pandemic, clinicians should strongly consider abdominal imaging in patients presenting with abdominal pain due to clostridium difficile infection in the setting of COVID-19 to rule out complications such as portal vein thrombosis. Early diagnosis and treatment of portal vein thrombosis prevent complications of portal hypertension and intestinal infarctions.

## 1. Introduction

About 18% of patients diagnosed with Coronavirus disease 2019 (COVID-19) present with gastrointestinal symptoms [1]. Common gastrointestinal manifestations include diarrhea, and abdominal pain and colitis may be observed in CT scans [2]. A high incidence of venous and arterial thrombotic complications has been observed in COVID-19 patients [3, 4]. Acute portal vein thrombosis (PVT) is a relatively uncommon complication in patients without malignancy or cirrhosis and is very rare in COVID-19 patients, with only three cases reported so far. Acute portal vein thrombosis is due to a combination of systemic and local risk factors for hypercoagulability. Clostridium difficile infection is not recognized as a risk factor for PVT. We present a unique case of acute PVT in a patient with Clostridium difficile infection in the setting of COVID-19 coagulopathy.

## 2. Case Presentation

A 66-year old female with a history of fibromyalgia, gastroesophageal reflux disorder, traumatic brain injury, anxiety, depression, hypertension, constipation, and acute blood loss anemia during recent spine surgery seven weeks ago was transferred to the emergency room (ER) from a rehabilitation facility for watery diarrhea of 10 days duration and altered mental status for one day. The patient was receiving physical therapy at the facility for the past five weeks following elective spine surgery involving posterior L3-S1 fusion for pseudoarthrosis. She received perioperative antibiotics. Her medications included duloxetine, lubiprostone, losartan, propranolol, and gabapentin.

She was diagnosed with SARS-CoV2 infection via nasopharyngeal swab testing at her facility during a routine screening two weeks before her presentation. She remained asymptomatic from a respiratory standpoint; however, she developed severe diarrhea and was receiving supportive treatment until she deteriorated.

**Figure 1:**
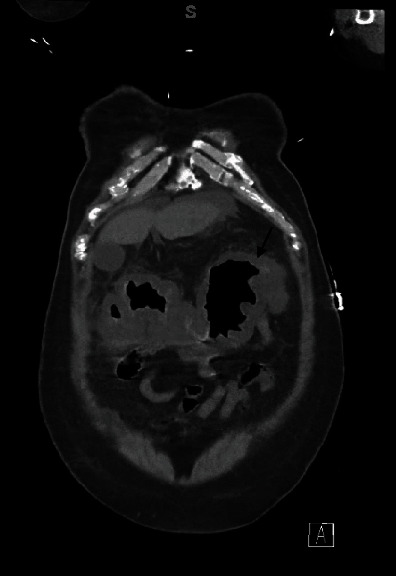
CT abdomen coronal view: arrow indicating thumbprinting sign indicative of colitis in a patient having coinfection with COVID-19 and Clostridium difficile.

In the ER, vitals were remarkable for hypotension with blood pressure 70/50 mmHg. Labs showed elevated INR, elevated creatinine, leukocytosis, and low serum bicarbonate (see Table 1). The abdomen was soft but had mild diffuse tenderness on palpation. She was hospitalized and underwent a CT scan of the head, chest, abdomen, and spine. CT abdomen revealed diffuse pancolitis (see Figure 1). Stool tested positive for Clostridium difficile infection, and she was started on oral vancomycin. A nasopharyngeal swab was positive for COVID-19. She was treated for sepsis due to Clostridium difficile infection, acute kidney injury, and acute encephalopathy. The patient's vitals and mental status improved to normal with fluid resuscitation and supportive management. She was treated with heparin for primary prevention of deep vein thrombosis. On Day 5, she developed a new-onset colicky and severe abdominal pain.

**Figure 2:**
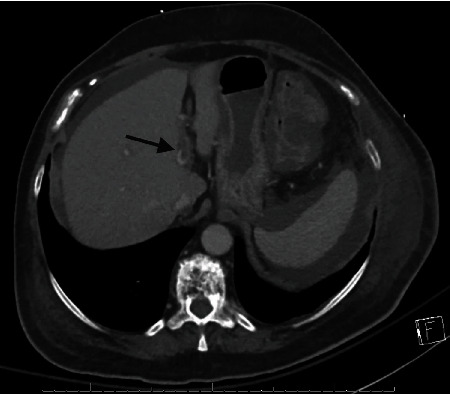
CT abdomen axial view: arrow indicating centrally located filling defect in the left portal vein suggestive of acute thrombus.

**Figure 3:**
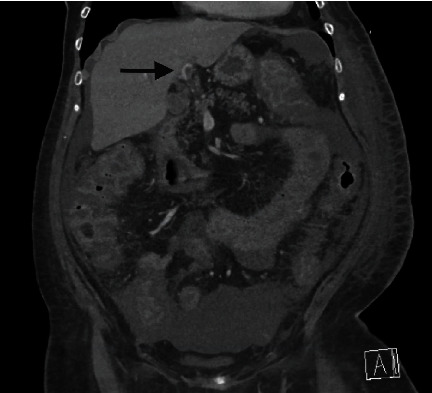
CT abdomen coronal view: arrow demonstrating acute left portal vein thrombosis.

Due to a lack of improvement in watery diarrhea, the persistence of abdominal pain, and the development of mild abdominal distension, a repeat CT scan of the abdomen/pelvis was obtained on the sixth day of the hospital course. It showed acute portal vein thrombus involving the left branch, moderate ascites along with findings of persistent colitis (Figures 2 and 3). Hepatic steatosis was identified, and there was no evidence of malignancy. JAK-2 mutation testing was negative. The patient was initially treated with intravenous unfractionated heparin and transitioned to apixaban at the time of discharge to complete six months of therapy. Her symptoms of diarrhea and abdominal pain resolved gradually in the next few days. Renal function returned to baseline. She was discharged back to the rehabilitation facility in a stable condition.

## 3. Discussion

Portal vein thrombosis (PVT) is caused when there is a partial or complete occlusion of the portal vein by a thrombus. Patients present with sudden onset severe colicky abdominal pain. Acute PVT results from a combination of local and systemic prothrombotic risk factors [5]. In adults, it typically occurs in patients with cirrhosis and hepatobiliary malignancy [6]. Other common local risk factors include inflammation of the pancreas or appendix, intra-abdominal infections, surgical injury to the portal venous system, and cancers involving the abdominal organs [7]. In most noncirrhotic and nonmalignant PVT, a systemic thrombophilic risk factor can be identified, but it is often multifactorial. They are most commonly caused by myeloproliferative disorders, antiphospholipid syndrome, or other hypercoagulable disorders [8]. Three cases of portal vein thrombosis in COVID-19 patients have been reported [3, 9, 10].

It is well established that COVID-19 is associated with an increased risk of arterial thrombosis and venous thromboembolism [4, 11]. In our patient with no prior history of thromboembolism, cirrhosis, malignancy, and negative JAK-2 V1617F mutation (myeloproliferative disorder), a combination of local risk factors (Clostridium difficile colitis and COVID-19 colitis) and systemic risk factor (COVID-19 coagulopathy) were likely responsible for the development of acute portal vein thrombosis.

Intra-abdominal infections are known risk factors for PVT. However, Clostridium difficile has not been recognized as a risk factor for PVT before. Although PVT in COVID-19 patients was mentioned in a few cases, the unique presentation of PVT in a COVID-19 patient with Clostridium difficile colitis has not been reported previously to our knowledge. Our case report adds valuable new information to the practicing clinicians. In view of the current COVID-19 pandemic, portal vein thrombosis should be considered in patients presenting with abdominal pain in the setting of diarrhea, especially in patients with additional local risk factors such as Clostridium difficile infection. Early diagnosis and treatment of PVT with vitamin K antagonists or direct oral anticoagulants has the potential to reduce morbidity and mortality [12]. Noncirrhotic acute PVT can lead to portal hypertension, and early anticoagulation prevents complications of portal hypertension and intestinal infarction [7]. More studies are needed to clarify the association between portal vein thrombosis and Clostridium difficile in COVID-19 patients.

## Figures and Tables

**Table 1:** Labs on admission.

WBC	31.9 K/mm^3^ (normal 4-11)
Hemoglobin	10.2 g/dL (normal 12-16)
Platelet	391 M/cc (normal 130-450)
INR	1.4
Creatinine	3.97 mg/dL (normal 0.6-1.40)
Serum bicarbonate	14 mmol/L (normal 19-31)
Lipase	6 IU (normal <65)
AST	23 U/L (normal 10-41)
ALT	12 U/L (normal 5-46)
Alk Phos	135 U/L (normal 42-146)
Ferritin	590 ng/mL (normal 14-313)
Procalcitonin	2.68 ng/mL (normal <=0.49)
C.Diff toxin gene by PCR	Positive
SARS-CoV-2 RNA RT-PCR (COVID-19)	Positive

## Data Availability

The data used to support the findings of this case report are included in the article.

## Abbreviations

COVID-19: Coronavirus disease 2019
PVT: Portal vein thrombosis
JAK: Janus kinase.
